# Spectrophotometric Analysis of Caffeine

**DOI:** 10.1155/2015/170239

**Published:** 2015-10-29

**Authors:** Showkat Ahmad Bhawani, Sim Siong Fong, Mohamad Nasir Mohamad Ibrahim

**Affiliations:** ^1^Department of Chemistry, Faculty of Resource Science and Technology, Universiti Malaysia Sarawak (UNIMAS), 94300 Sarawak, Malaysia; ^2^School of Chemical Sciences, Universiti Sains Malaysia, 11800 Pulau Pinang, Malaysia

## Abstract

The nature of caffeine reveals that it is a bitter white crystalline alkaloid. It is a common ingredient in a variety of drinks (soft and energy drinks) and is also used in combination with various medicines. In order to maintain the optimum level of caffeine, various spectrophotometric methods have been developed. The monitoring of caffeine is very important aspect because of its consumption in higher doses that can lead to various physiological disorders. This paper incorporates various spectrophotometric methods used in the analysis of caffeine in various environmental samples such as pharmaceuticals, soft and energy drinks, tea, and coffee. A range of spectrophotometric methodologies including chemometric techniques and derivatization of spectra have been used to analyse the caffeine.

## 1. Introduction

**Figure 1:**
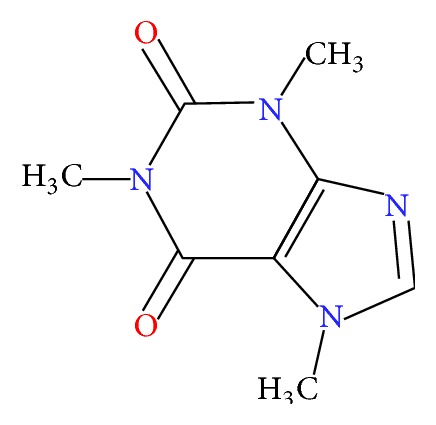
Structure of caffeine.

As we know, caffeine (Figure 1) is the most versatile compound in the sense that almost every human being is exposed to this compound via various beverages and medicines. Caffeine is widely used in many soft drinks as flavouring agent and is deliberately added to make people addicted to these drinks. Caffeine is a naturally occurring alkaloid and it can be found in at least 63 plant species and is present in their leaves, seeds, and fruits [1]. The amount of caffeine varies according to species and origin of plants [2]. Caffeine belongs to the family of naturally occurring powerful xanthines and possibly the oldest known stimulants. Therefore, this property exhibits its ability to provide alertness, put off sleep, and increases the alertness in the study [3].

It is a well-established fact that caffeine acts as a stimulant to the central nervous system and heart and also increases the activity of brain through its adenosine antagonist action. Nowadays, it is most commonly used in various pharmaceuticals. Caffeine is used in the treatment of mild respiratory depression caused by narcotics and for the treatment of circulatory failure [4]. It is used with aspirin in some preparations for the treatment of headache and with ergotamine in antimigraine preparations in order to produce a sense of alertness [5].

The determination of caffeine in various natural products is also very important aspect from an economic point. Decaffeination of various natural products provides a valuable byproduct such as caffeine and that can be used in preparation of various drugs.

It is a well-established fact that the spectrophotometric determination in UV-vis region is less expensive, follows a simple procedure, and provides a high accuracy and reproducibility from a small number of samples. Spectrophotometry is widely used in all the schools, colleges, universities, and research institutes. Almost all the researchers are capable of handling this instrument. A wide variety of sophisticated instruments are available such as HPLC [6–8] and GC [9–11] and are frequently used for the analysis of caffeine. But every researcher is not able to access these sophisticated instruments. The contents of this review will boost the knowledge of the researchers working on caffeine in small scale industries, colleges, and universities.

## 2. Different Validation Methods for Quantification of Caffeine

**Figure 2:**
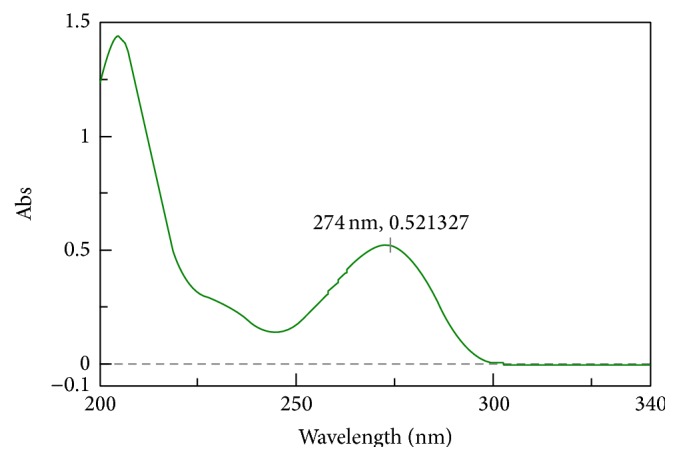
Spectrum of caffeine.

Spectrophotometric measurement is the most popular analytical tool in the field of analysis of a variety of compounds in simple as well as in complex mixtures. On the other hand, various modifications in the software of spectrophotometers lead to multicomponent analysis. The derivatization especially has resolved the main drawback of this technique by resolving the spectra of complex matrix into its individual components. This paper deals with the identification and estimation of caffeine by spectrophotometry. A list of various methodologies are presented in Table 1. The data presented in Table 1 provides an idea especially about the linearity and spectral ranges for the scanning and analysis of caffeine by different methodologies. It is more clear that researchers are devoted to provide new ideas for the modification of spectrophotometry in the analysis of caffeine. Some researchers are focused on the utilization of chemometric techniques while others are more interested in derivatization of spectra. Simple spectrum of caffeine in distilled water is presented in Figure 2.

### 2.1. Spectrophotometric Determination of Caffeine in Pharmaceuticals

Two chemometric calibration techniques such as inverse least squares (ILS) and principal component analysis (PCA) or (factor based) have been used for the spectrophotometric determination of metamizol, acetaminophen, and caffeine in pharmaceuticals [12]. In this study MAPLE software was used for the calculations. All the measurements were carried out in the spectral range from 225 to 285 nm in the intervals of Δ*λ* = 5 nm at 13 wavelengths in the zero-order spectra.

#### 2.1.1. Methods

*(1) Inverse Least Squares [6]*. It is the inverse expression of Beer-Lambert law:

(1) $$\begin{array}{c} C=P\times A. \end{array}$$

This equation can be expressed as a linear equation system as follows:

(2) C1=P11A1+P12A2+⋯+P1wAw,C2=P21A1+P22A2+⋯+P2wAw,C3=P31A1+P32A2+⋯+P3wAw,Cc=Pc1A1+Pw2A2+⋯+PcwAw.

*A*_*w*_ represents the absorbance at *w*th wavelength, *P*_*w*_ represents the calibration coefficient for *C*th component at *w*th wavelength, and *C*_*c*_ is the concentration of *C*th component.

*(2) Principal Component Analysis [12]*. This model is expressed by the following equation:

(3) $$\begin{array}{c} A_{proj}={V_{C}}^{T}A, \end{array}$$

where *A*_proj_ represents the matrix containing new coordinates, *A* represents the original training set absorbance matrix, and *V*_*C*_^*T*^ containing the basis vectors. Consider

(4) $$\begin{array}{c} C=FA_{prog}, \end{array}$$

where *F* represents the calibration coefficient for the obtained linear equation system.

The PCA is evaluated in two steps: first step involves the determination of eigenvectors or factors for absorbance data and the second step uses MLR to regress the concentration data matrix. The mean recoveries and relative standard deviation for the CAF, MET, and ACE were found to be more than 99% and less 2%, respectively.

The simultaneous determination of phenylpropanolamine hydrochloride (I), caffeine (II), and diazepam (III) in tablets was performed by a reliable and specific UV spectrophotometric method [13]. This method was validated and compared with a liquid chromatographic method. The developed method was rapid, cost-effective, and easy to perform. The LOD and LOQ obtained for these three components were 0.049 and 0.16 mg/mL (I), 1.86 × 10^−4^ and 8.4 × 10^−4 ^mg/mL (II), and 3.08 × 10^−3^ and 10 × 10^−3 ^mg/mL (III). The recoveries for all the components, I, II and III, were >98.04%.

Dinç et al. [14] have used three methods for the determination of chlorphenoxamine hydrochloride (CP) and caffeine (CAF) in the formulated mixture. From the first method analytical signals were measured for both drugs at wavelengths corresponding to either maxima and minima in the first derivative spectra of the ratio spectra. And from the other two methods (chemometric techniques), the absorbance data corresponding to the concentration data was obtained by measurements in the range 225–285 nm. The values of SEP completely acceptable were 0.28 and 0.59 (CP) and 0.48 and 0.40 (CAF), respectively, for CLS and ILS methods. Similar results were obtained for the SEC and the values found acceptable were 0.31 and 0.65 (CP) and 0.53 and 0.44 (CAF), respectively, for CLS and ILS methods. The mean recoveries and RSD were found to be 101.4, 1.40 and 101.8, 1.79% (CP), and 99.1, 1.68, and 99.2, 1.56% (CAF), respectively, for CLS and ILS techniques.

The expression for the classical least-squares (CLS) is expressed as follows.

The equation for the CLS [8] can be written as

(5) $$\begin{array}{c} A=K\times C. \end{array}$$

CLS method is actually application of MLR to the classical expression of Beer-Lambert law.

This equation can be written in a linear equation system as follows:

(6) A1=K11C1+K12C2+⋯+K1cCc,A2=K21C1+K22C2+⋯+K2cCc,A3=K31C1+K32C2+⋯+K3cCc,Aw=Kw1C1+Kw2C2+⋯+KwcCc,

where *A*_*w*_ is the absorbance at *w*th wavelength, *P*_*cw*_ is the calibration coefficient for *C*th component at *w*th wavelengths, and *C*_*c*_ is the concentration of *C*th component.

An N-PLS method was adopted by Sena and Poppi [15] for the simultaneous determination of ASA, PRC, and CAF in pharmaceutical formulations. In this procedure the calibration set was constructed with nine solutions with different concentrations at four different pH values, 2.0, 3.0, 4.0, and 5.0. The best model obtained by PLS was at pH 5.0. The better results were achieved by an N-way PLS model applied to a three-way array at all PH data sets. The RMSEP obtained were from 11.5–35% lower than those obtained with PLS at pH 5.0. The recoveries of these analytes from tablets were in the range of 94.7–104.5%.

The quantitative abilities of multivariate calibration methods (PLS-1 and PCR) were compared at absorption (zero-order) spectra and first-order derivative spectra for the determination of phenytoin, barbital, and caffeine [16]. It was found that both the approaches were statistically applied for their determination in synthetic and formulated mixtures. But the significant results were achieved by using the first-order spectra. The relative standard errors for these determinations were less than 3% in most cases.

Ashour et al. [17] proposed two spectrophotometric methods for the simultaneous determination of paracetamol and caffeine from pharmaceuticals and their synthetic mixtures. These methods are based on the application of continuous wave transform (CWT) and derivation transform on ratio spectra. Authors have tested several wavelet families but Coif1 and Sym2 were found to be best under optimum conditions.

Dinç et al. [18] introduced a derivative ratio spectra-zero crossing spectrophotometry for determination of paracetamol, propyphenazone, and caffeine in ternary mixture. This method is based on the simultaneous use of first derivative of ratio spectra and measurements of derivative ratio analytical signals corresponding to the zero crossing points of wavelengths. In this work authors have used first propyphenazone as a division for the determination of paracetamol and caffeine by measuring first derivative ratio at 242.8 nm (zero crossing for caffeine) and 251.2 and 273.8 nm (zero crossing for paracetamol). And for the determination of contents of propyphenazone and caffeine in the same ternary mixture paracetamol was used as a division and the first derivative was measured at 244.8 and 276.9 nm (zero crossing for caffeine) and 250.6 and 274.0 nm (zero crossing for propyphenazone), respectively. The data obtained from the results suggested that the developed method is useful for the analysis of the synthetic ternary mixtures and tablets containing PAR, PAZ, and CAF. It has been observed that when PRO was used as a division, the mean recoveries and RSD were found to be 100.2 and 0.64% for pAR and 99.6 and 0.93% for CAF. And when PAR was used as division the mean recoveries and RSD were found to be 99.2 and 1.54% for PRO and 99.5 and 1.05% for CAF.

Vichare et al. [19] have developed two UV spectrophotometric methods for the estimation of caffeine concentration in a drug containing caffeine and paracetamol. In this study first method involved the simultaneous equation method and absorption of caffeine was recorded at 273 nm (*λ*_max_), while the other method involves the formation of Q-absorbance equation at isosbestic point at 259.5 nm. From both methods linearity concentration range was 2–2.3 *μ*g/mL for caffeine. The credibility of this work was validated on the basis of %RSD which was found to be less than 2 and coefficient of correlation close to 1.

Somya et al. [20] have estimated caffeine in an injection containing sodium benzoate by isoabsorption method (isosbestic method). The absorption ratio method was applied for the estimation of concentration of caffeine and sodium benzoate. In this study the isosbestic point was observed at 242 nm. Bharate et al. [21] have estimated caffeine and acetylsalicylic acid in both pure and tablet dosage form. The findings of this study are based on two methods; first one is the simultaneous equation method and the second one is absorption ratio method. All the studies were carried out in 0.1 N NaOH solution. In simultaneous equation method the absorbance maximum was recorded at 297 and 272 nm for acetylsalicylic acid and caffeine, respectively, while the measurements involved in absorption ratio method were determined at isoabsorption point at 289 nm. In both cases the linearity concentration range for caffeine was 0–25 *μ*g/mL. In another study S. S. Bharate and S. B. Bharate [22] have used 0.1 N HCl instead of 0.1 N NaOH for the estimation of caffeine by above two mentioned methods.

A comparative study has been performed by Khoshayand et al. [23] by using different chemometric methods such as partial least square regression (PLS), genetic algorithm coupled with PLS (GA-PLS), and principal component-artificial neural network (PC-ANN) for the determination of Paracetamol, ibuprofen, and caffeine in pharmaceuticals. From their whole study, it is concluded that on the basis of analytical performance the GA-PLS shows superiority over the other methods. The reason for the superiority was due to the wavelength selection in PLC calibration using genetic algorithm without loss of prediction capacity.

A simple kinetic standard H-point spectrophotometric method was used for the simultaneous determination of paracetamol and caffeine [24]. This method is based on the reaction of Cu(II) with paracetamol and caffeine in the presence of neocuproine (Nc) and SDS in buffer solution. The following complex reaction was proposed as follows:

(7) $$\begin{array}{c} n{Cu}^{2+}+2nNc+A_{red}⟷n{\left[\;Cu{\left(\;Nc\;\right)}_{2}\;\right]}^{+}+A_{ox} \end{array}$$

The H-point standard addition method is actually the modification of the standard addition method and permits direct correction of both proportional and constant errors produced by sample matrix. In this study various experimental conditions have been optimized. And also this method has been applied in the analysis of paracetamol and caffeine in various synthetic materials.

Singh and Sahu [25] have developed a method for the determination of caffeine based on the oxidation of caffeine with sodium metaperiodate in the presence of acetic acid. This is followed by coupling with 3-methyl-2-benzothiazoline hydrazone hydrochloride (MBTH) which results in a blue colored product with *λ*_max_ at 630 nm. This method was applied in the determination of caffeine in pure alkaloids and in pharmaceutical formulations.

A derivative spectrophotometric method was developed for the determination of caffeine in foods and also in pharmaceuticals containing antihistamines [26]. In this study an ethanolic solution of mixture containing caffeine and chlorpheniramine maleate that was subjected to UV-scanning showed a band overlap of the maxima at 273 nm for caffeine and 262 nm for chlorpheniramine maleate in the zero-order (*D*_0_), while the first derivative scanning of some binary mixtures shows a trough for caffeine at 288 nm and zero absorbance for chlorpheniramine at this wavelength. The same case was observed for caffeine when chlorpheniramine produces a trough at about 273 nm and caffeine reads zero absorbance.

### 2.2. Spectrophotometric Determination of Caffeine in Beverages

It is very important aspect to estimate the content of caffeine in various beverages. The second- and third-order derivative spectrophotometric method was used for the determination of caffeine in cola, coffee, and tea [27]. This method was applied without any separation and background correction technique or reagent. Pelozo et al. [28] have estimated caffeine and various polyphenols in the seeds of* Paullinia cupana* var.* sorbilis*. The quantification was performed in the extractive solution and in the granulated form of seeds. The linearity range for caffeine was found to be 5–25 *μ*g/mL.

Various spectrophotometric methods have been adopted for the estimation of caffeine in various brands of tea, coffee, and so forth [29]. Wanyika et al. have determined the content of caffeine in tea and instant coffee brands available in Kenyan market [1]. Khanchi et al. [30] established an artificial neural networks for the prediction of concentration of caffeine and theobromine in four Iranian tea samples. The content of caffeine was estimated in different brands of coffee based on the quantification by standard addition method [31]. The amount of caffeine was influenced by the temperature of water used to brew, and the time of brewing and was independent of volume of water used for brewing. Belay et al. [32] have opened a new window for the estimation of caffeine in coffee beans. This method was based on the measurement of molar decadic absorption coefficients and transitional dipole moment of pure caffeine in water and dichloromethane. The molar decadic absorption coefficient in water and dichloromethane were found to be 1115 and 1010 m^2 ^mol^−1^, respectively, and these were obtained at 272 and 274.7 nm. The transitional dipole moment of caffeine in water and in dichloromethane was 10.40 × 10^−20^ and 10.80 × 10^−30^, respectively.

The characterization of pure caffeine and the determination of amount of caffeine present in twelve tea brands was performed on the basis of optical transition properties of caffeine in different solutions such as dichloromethane, water, chloroform, and ethyl acetate [33]. The outcome of the study reported that caffeine have optical transition in dichloromethane as compared to other solvents. Maidon et al. [34] have also used different solvents such as water, ethyl acetate, chloroform, and methanol for the spectrophotometric determination of caffeine in tea leaves. Based on their study, chloroform was found to be best for the determination of caffeine. A simple spectrophotometric method was adopted by Amos-Tautua et al. [35] in which CCl_4_ was used as an extracting solvent for the determination of caffeine in various soft and energy drinks.

## 3. Conclusion

Spectrophotometry as an analytical tool for the determination of caffeine in various kinds of samples is very simple and is accessible to everyone. Various chemometric methods have been applied in the determination of content of caffeine. These different chemometric methods have increased the scope of spectrophotometry. Many complicated spectrum related errors have been minimised by derivatization of spectra. The reason for the interferences is because a recorded spectrum is sum of absorption of analyte and matrix. In case of simple samples this can be corrected by omitting the background measurements versus blank. On the other hand for the complex samples selectivity can be increased by derivatization of spectra. Derivatization removes the spectral interferences and provides a better selectivity.

## Figures and Tables

**Table 1:** Different spectrophotometric methods for the determination of caffeine.

S. number	Method	Linearity range for caffeine	UV-vis spectral range	Sample	Solution	Reference
1	Chemometric methods for spectral investigation (1) Inverse least square (2) Principal component analysis	12–56 *μ*g/mL	225–285 nm	Pharmaceuticals (Metamizol, acetaminophen, and caffeine)	0.1 M HCl	[12]

2	Simple and derivative spectrophotometry	12–28 *μ*g/mL	200–350 nm	Pharmaceuticals (Phenylpropanolamine HCl, caffeine, and diazepam)	Water, chloroform, and petroleum ether	[13]

3	Derivative ratio spectra-zero crossing procedure	1–5 *μ*g/mL	244.8–276.9 nm	Pharmaceuticals (Paracetamol, propyphenazone, and caffeine)	0.1 M HCl	[18]

4	Ratio spectra spectrophotometry and chemometric methods viz, classical least squares, and inverse least squares	4–40 *μ*g/mL	225–285 nm	Pharmaceuticals (Chlorphenoxamine hydrochloride and caffeine)	0.1 M HCl	[14]

5	Multivariate calibration and N-way partial least squares (PLS)	2–6 *μ*g/mL	210–300 nm	Pharmaceuticals (Acetylsalicylic acid, paracetamol, and caffeine)	Water	[15]

6	Multivariate calibration method and chemometric methods viz, partial least squares, and principle component regression	.05–20 *μ*g/mL	190–300 nm	Pharmaceuticals (Phenytoin, barbital, and caffeine)	Water/methanol (1 : 1, v/v)	[16]

7	Continuous wavelet transform and derivative transform (using Savitzky-Golay filters)	2–50 *μ*g/mL	220–300	Pharmaceuticals (Paracetamol and caffeine)	0.1 M HCl	[17]

8	H-point standard addition method	0.1–3.0 *μ*g/mL	453 nm	Pharmaceuticals (Paracetamol and caffeine)	Water	[24]

9	Simultaneous equation method and Q-absorbance equation at isosbestic point	2–32 *μ*g/mL	200–400 nm	Pharmaceuticals (Paracetamol and caffeine)	Water	[19]

10	Isoabsorption assay method	—	200–300 nm	Pharmaceuticals (Caffeine and sodium benzoate)	Water	[20]

11	Simultaneous equation method and absorbance ratio method	0–25 *μ*g/mL	200–400 nm	Pharmaceuticals (Acetylsalicylic acid and caffeine)	0.1 N NaOH	[21]

12	Simultaneous equation method and absorbance ratio method	1.14–2.05 *μ*g/mL	200–400 nm	Pharmaceuticals (Acetylsalicylic acid and caffeine)	0.1 N HCl	[22]

13	Partial least squares regression, genetic algorithm coupled with PLS, and principle component-artificial neural network	1–18 *μ*g/mL	200–400 nm	Pharmaceuticals (Paracetamol, ibuprofen, and caffeine)	Methanol/0.1 N HCl (3 : 1, v/v)	[23]

14	Simple spectrophotometric method with coupling reagent	0.1–1 *μ*g/mL	500–750 nm	Alkaloids (caffeine and theophylline)	Water	[25]

15	First-derivative spectrophotometry	4–40 *μ*g/mL	220–360 nm	Pharmaceuticals (Chlorpheniramine maleate and caffeine)	Ethanol	[26]

16	Derivative spectrophotometric methods (first, second, and third-order spectra)	2–10 *μ*g/mL	190–350 nm	Beverages (caffeine)	Water	[27]

17	Simple spectrophotometric method	5–25 *μ*g/mL	271 nm	*Paullinia cupana *var*. sorbilis *(tannins and caffeine)	Sulfuric acid (2.5%)	[28]

18	Simple spectrophotometric method (Solvent study)	0–20 *μ*g/mL	180–400 nm	Tea (caffeine)	Water, ethyl acetate, chloroform, and methanol	[34]

19	UV-spectrophotometry	10–60 *μ*g/mL	200–600	Soft and energy drinks (caffeine)	Carbon tetrachloride	[35]

20	Simple spectrophotometric method	10–50 *μ*g/mL	200–400 nm	Drugs (caffeine)	Water	[36]
